# Supplementary figures and images for: Pre‐vaccination immunotypes reveal weak and robust antibody responders to influenza vaccination
Source: Aging Cell. 2023 Dec 25;23(2):e14048. doi: 10.1111/acel.14048 (PMC10861208; doi:10.1111/acel.14048)

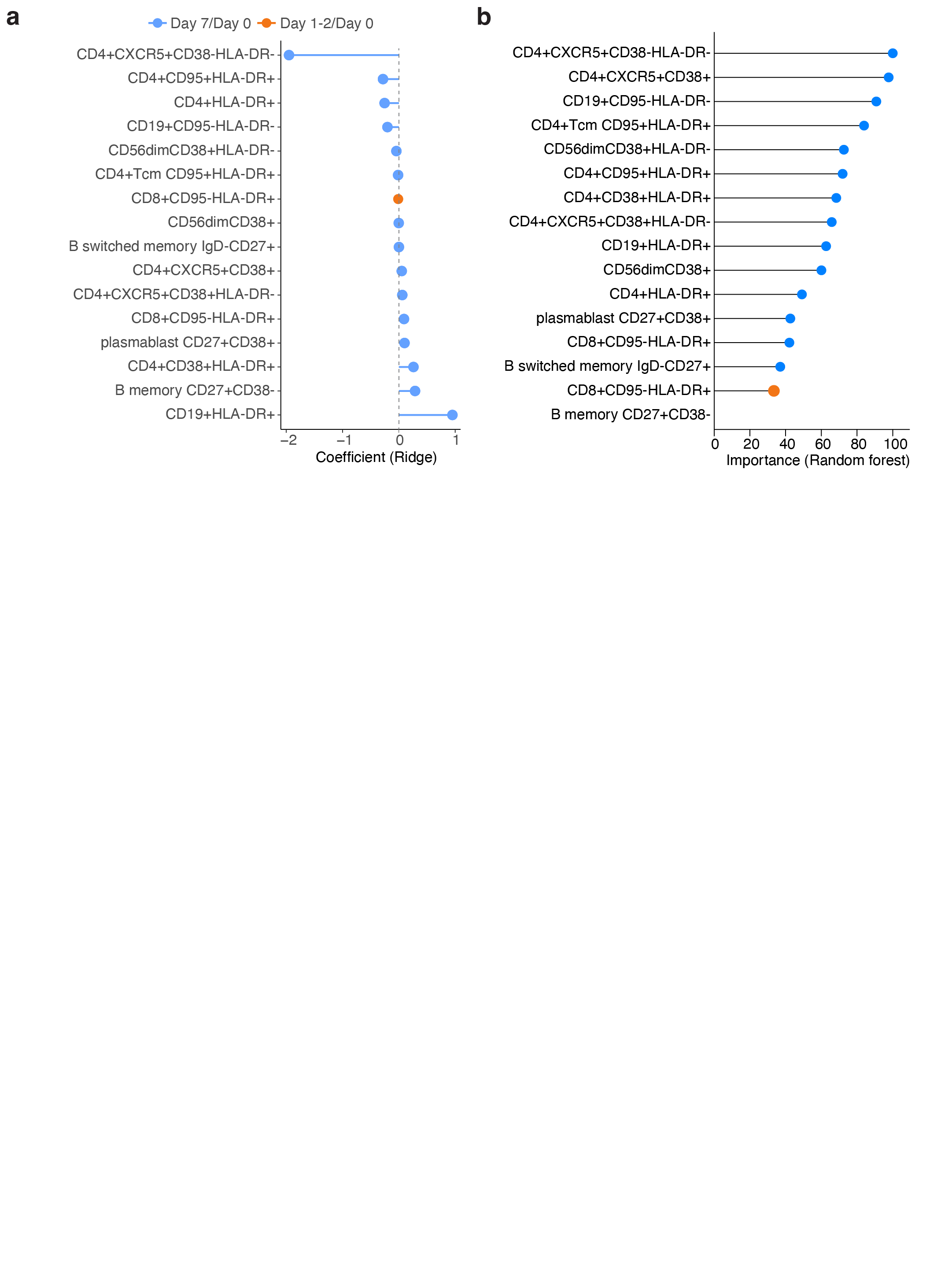

Supplement: Supplementary file 1 — Figure S1: Flowchart of data analyses. Figure S2: Percentage of nonactivated follicular CD4+ T cell increase at day 7 is the most important associate of antibody responses in ridge & random forest regression models. (a) Coefficients (Ridge regression) and (b) variable importance (random forest regression) of day 28 HI titer regression analyses. Percentages of significant immune subsets (adj.p < 0.05) that associated with antibody responses are used in both regression analyses. [file ACEL-23-e14048-s001.zip › Figure_S2.png]

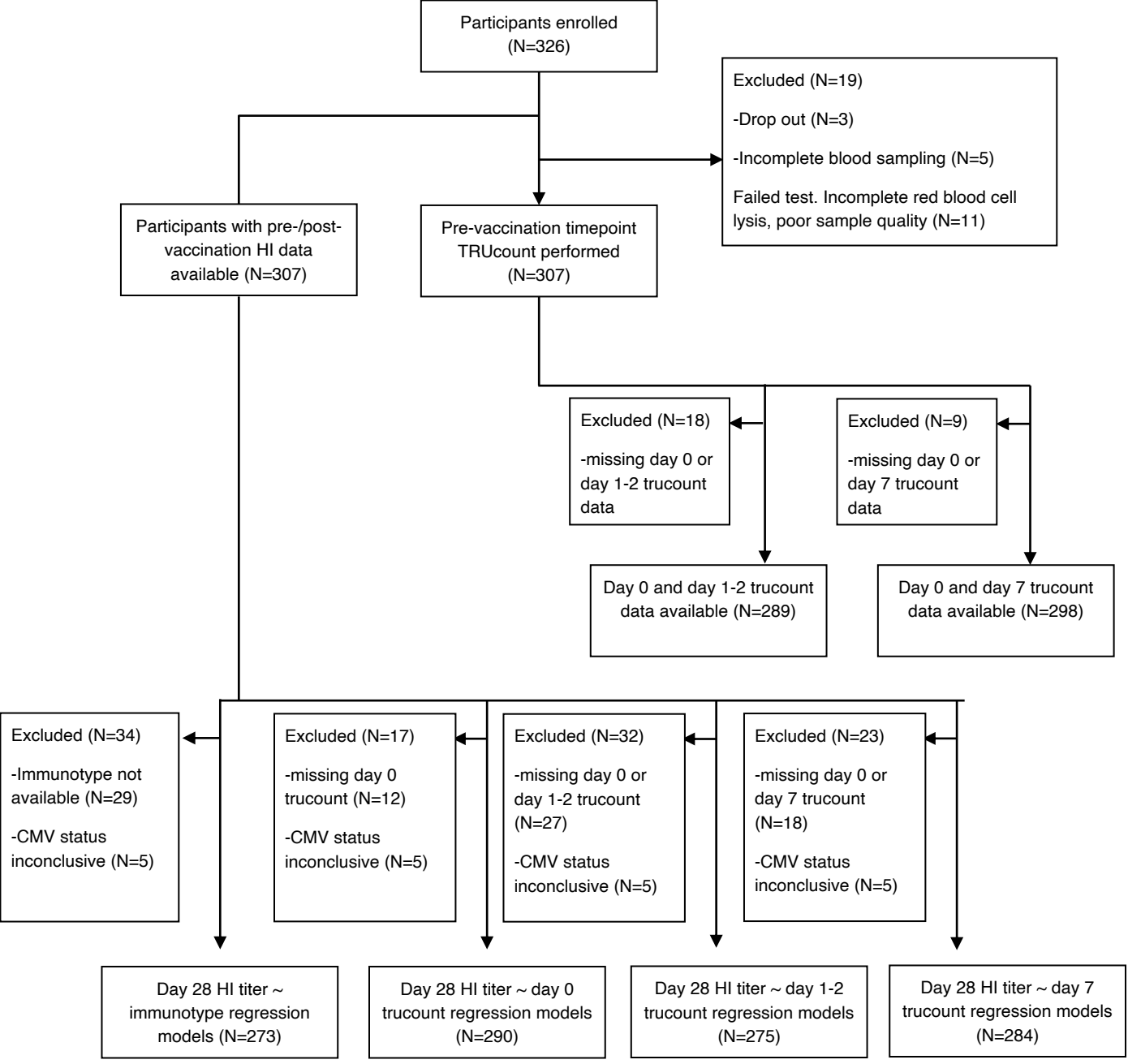

Supplement: Supplementary file 1 — Figure S1: Flowchart of data analyses. Figure S2: Percentage of nonactivated follicular CD4+ T cell increase at day 7 is the most important associate of antibody responses in ridge & random forest regression models. (a) Coefficients (Ridge regression) and (b) variable importance (random forest regression) of day 28 HI titer regression analyses. Percentages of significant immune subsets (adj.p < 0.05) that associated with antibody responses are used in both regression analyses. [file ACEL-23-e14048-s001.zip › Figure_S1.pdf]
